# Supplementary material for: Hybrid Living Capsules Autonomously Produced by Engineered Bacteria
Source: Adv Sci (Weinh). 2021 May 3;8(11):2004699. doi: 10.1002/advs.202004699 (PMC8188213; doi:10.1002/advs.202004699)
Supplement: Supplementary file 1 — Supporting Information [file ADVS-8-2004699-s001.pdf]

© 2021 Wiley-VCH GmbH

Supporting Information

for *Adv. Sci.,* DOI: 10.1002/advs.202004699

Hybrid Living Capsules Autonomously Produced by Engineered Bacteria

*Daniel P. Birnbaum, Avinash Manjula-Basavanna, Anton Kan, Blaise L. Tardy, Neel S. Joshi**

Supporting Information

Hybrid Living Capsules Autonomously Produced by Engineered Bacteria

*Daniel P. Birnbaum, Avinash Manjula-Basavanna, Anton Kan, Blaise L. Tardy, Neel S. Joshi**

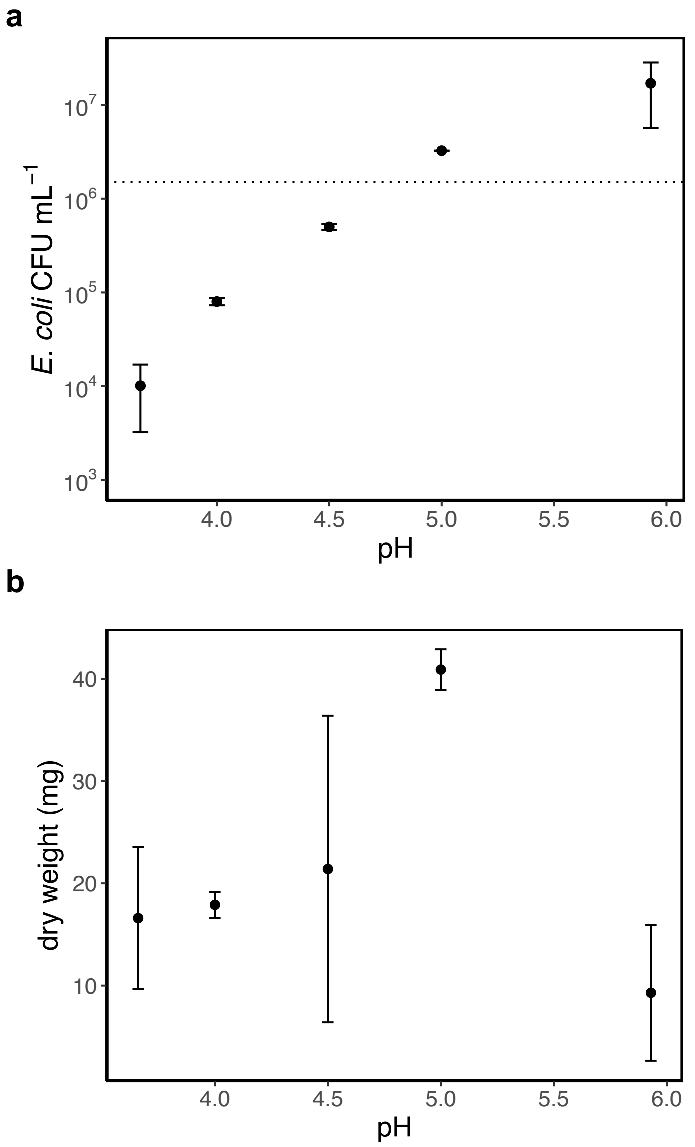

Figure S1. Screen to determine optimal pH of HS media for co-culture. (a) 10 mL HS media in a Falcon tube was inoculated with *G. hansenii* and *E. coli*. The pH of the HS media was pre-adjusted to various levels using citric acid (unadjusted HS media had pH of 5.9). After 5 days of incubation, the *E. coli* concentration in the liquid underlying the cellulose pellicle was measured by plating on selective LB-agar. The dotted line indicates the initial concentration of *E. coli.* Values and error

bars reflect the mean ± s.d. of three biological replicates (n = 3). (b) Dry weight of cellulose pellicles following the 5-day co-culture period. Values and error bars reflect the mean ± s.d. of three biological replicates (n = 3).

Efficiency of *E. coli* encapsulation

As noted in the main text, we observed that the capsules usually produced a turbid culture during the incubation in LB, due to *E. coli* cells escaping the cellulose matrix. However, certain capsules which appeared to only contain *E. coli* colonies in the core of the cellulose matrix—such as those pictured in Figure 3A inoculated with 10% *G. hansenii* and grown for 4 or 5 days in HS—did not produce turbid cultures. This suggests that *E. coli* which escape into the surrounding LB originate primarily from surface-level colonies, such as the one imaged in Figure 4C.

To evaluate the efficiency of *E. coli* encapsulation within the cellulose matrix, we produced capsules containing many surface-level colonies of BL21 / pBbA8k-RFP using the standard protocol described in the methods. Following the growth period in LB, the capsules were washed three times in 1 mL PBS and then incubated in 0.5 mL PBS in a 2 mL Eppendorf tube for 24 hours at room temperature with agitation (800 rpm, Eppendorf Thermomixer R) to determine how many cells would leak into the PBS supernatant vs. be stably retained in the capsule matrix. After the 24-hour incubation in PBS, the supernatant was removed and the capsule was degraded in PBS with mechanical homogenization and cellulase treatment as described in the methods. Finally, the supernatant and degradant were separately plated on selective LB-agar to obtain CFU counts

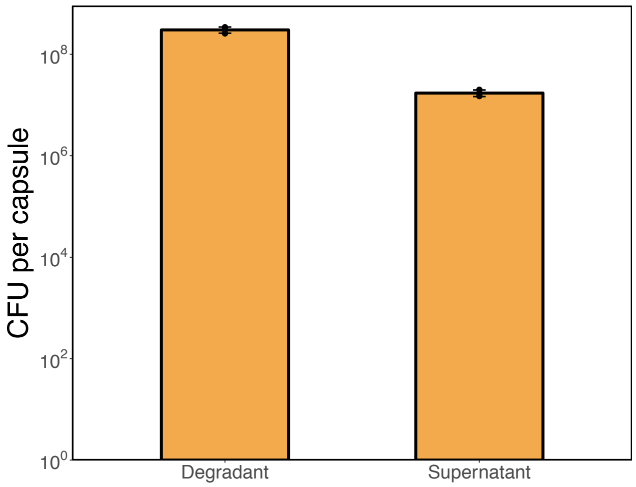

Figure S2. Efficiency of *E. coli* encapsulation. Values and error bars reflect the mean ± s.d. of three biological replicates (n = 3).

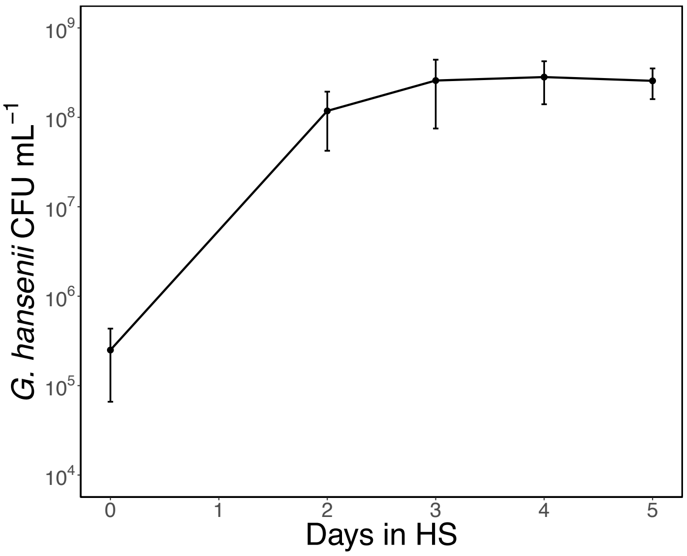

Figure S3. Time course of *G. hansenii* growth in monoculture capsules. After the incubation in HS, the capsules were degraded in PBS as described in methods, and plated on HS-agar to quantify *G. hansenii* CFU. Values and error bars reflect the mean ± s.d. of three biological replicates (n = 3).

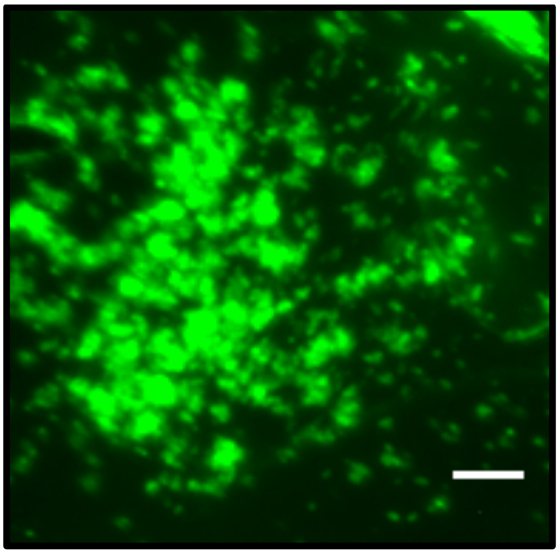

Figure S4. Cross-sectional image of capsule containing GFP sequestered by NbGFP-displaying curli fibers. Section was imaged using an EVOS fluorescent microscope with a GFP light cube (470/22 nm excitation; 525/50 nm emission). Scale bar = 10 µm.

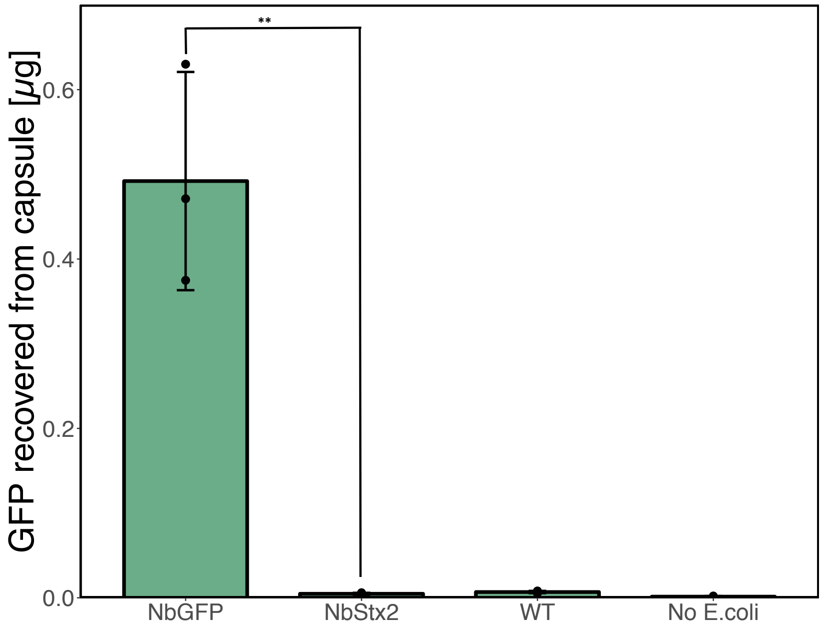

Figure S5. Recovery of sequestered GFP from capsules. Capsules containing sequestered GFP were washed and incubated in 1 M urea with agitation overnight (230 rpm) to disrupt the interaction between NbGFP and captured GFP. GFP fluorescence in the 1 M urea supernatant was then measured on a BioTek Synergy NEO plate reader (485 nm excitation, 428 nm emission) to estimate

the amount of GFP which had been released from the capsules. Fluorescence measurements were converted to GFP concentrations using a calibration curve of purified GFP in 1 M urea. Values and error bars reflect the mean ± s.d. of three biological replicates (n = 3). Significantly more GFP was recovered from capsules containing the NbGFP-displaying curli fibers compared to capsules containing NbStx2-displaying curli fibers (two-sided Student *t*-test for two means, P-value = 0.0028). ** indicates P-value < 0.01.

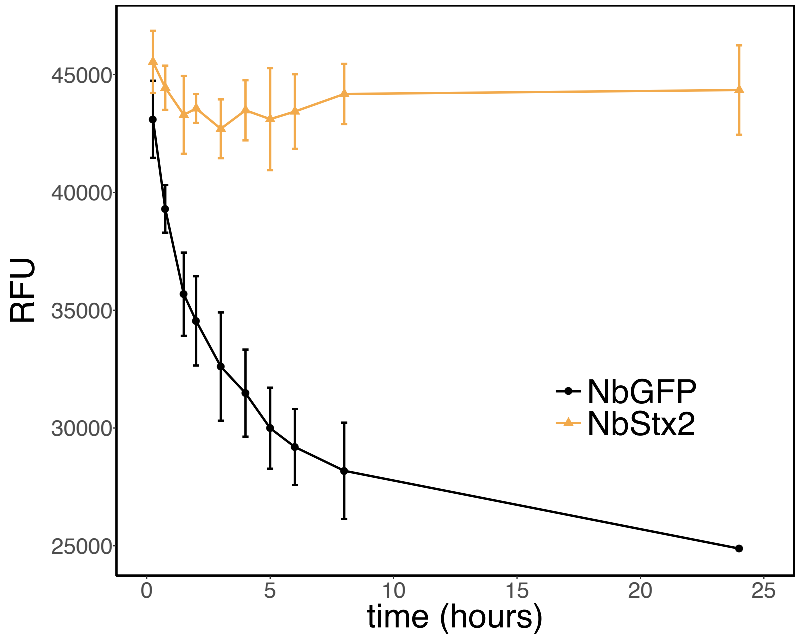

Figure S6. Kinetics of GFP sequestration in capsules. Capsules containing engineered curli fibers with NbGFP fusion domains (or NbStx2 in the negative control) were incubated 0.5 mL PBS containing 4.6 µg/mL purified GFP at 37 ºC with agitation. The supernatant of the solution was sampled with replacement at various time points and GFP fluorescence was measured on BioTek Synergy NEO plate reader (485 nm excitation, 428 nm emission). The fluorescence of the supernatant decreased for the NbGFP sample due to GFP being sequestered in the capsule. Values and error bars reflect the mean ± s.d. of three biological replicates (n = 3).

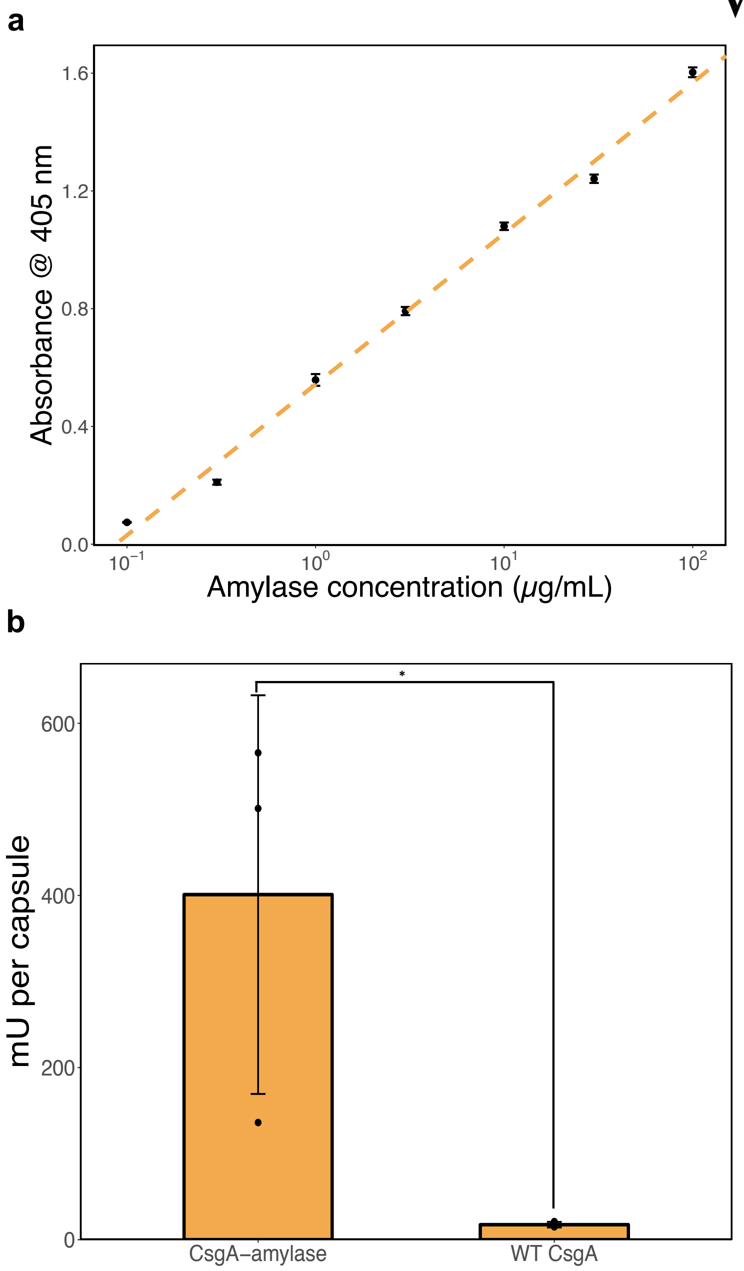

Figure S7. α-Amylase activity in capsules containing amylase-displaying curli fibers. (a) A calibration curve of purified α-amylase was computed by measuring the absorbance of solution at 405 nm (BioTek Synergy NEO) after co-incubation of amylase and 3 mg/mL chromogenic substrate 4-Nitrophenyl α-D-maltohexaoside under agitation at 37 ºC for 5 hours. Values and error bars reflect the mean ± s.d. of three biological replicates (n = 3). (b) Units of α-amylase activity per capsule containing either amylase-displaying or wild-type curli fibers were computed using the calibration curve above. Values and error bars reflect the mean ± s.d. of three biological replicates (n = 3). Capsules containing amylase-displaying fibers had significantly more units of α-amylase

activity (two-sided Student *t*-test for two means, P-value = 0.046). We assumed 500 U/mg of purified amylase for the calibration. * indicates P-value < 0.05.

Colonization of empty BC capsules by externally added *E. coli*

Given the ability of *E. coli* to escape the cellulose matrix of capsules produced via co-culture, we hypothesized that externally added *E. coli* may be able to colonize empty capsules (i.e. capsules produced from a monoculture of *G. hansenii*). To test this, we seeded empty capsules with *E. coli* by incubating them in LB inoculated with BL21 cells with plasmid pBbA8k-RFP until the culture reached late exponential phase (OD_{600} = 0.7). The capsules were then transferred back to PTFE to incubate at 37 ºC in humid conditions for 20 hours. Finally, the capsules were transferred to fresh 5 mL LB containing kanamycin (50 µg/mL) and 0.1% (w/w) arabinose, and incubated in a 50 mL Falcon tube at 37 ºC under shaking conditions for 20 hours (230 rpm). We found that during this final round of incubation in LB, the previously empty capsules exhibited moderate *E. coli* colony growth similar to capsules produced using the co-culture protocol outlined in the paper (Figure S8). This represents a possible alternative protocol to producing cellulose capsules incorporated with engineered *E. coli*.

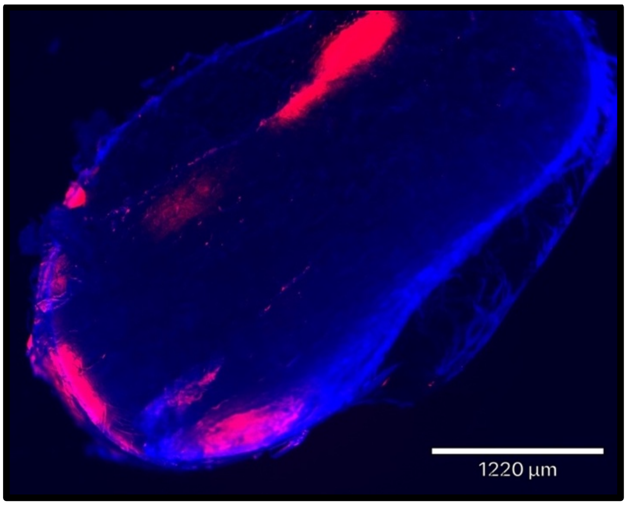

Figure S8. Cross sectional image of a capsule containing RFP-expressing *E. coli*. This capsule was produced by externally adding *E. coli* to an empty capsule produced from a monoculture of *G.*

hansenii, as described above. It was then stained with CFW, embedded in gelatin, and fixed in PFA as described in the methods before being sectioned with a razor blade. CFW fluorescence is shown in blue and RFP fluorescence is shown in red. The sections were imaged using an ECHO Revolve 4 fluorescent microscope with a DAPI light cube for CFW (385/30 nm excitation; 450/50 nm emission), and a TRITC light cube for mRFP1 (530/40 nm excitation; 590/50 nm emission).

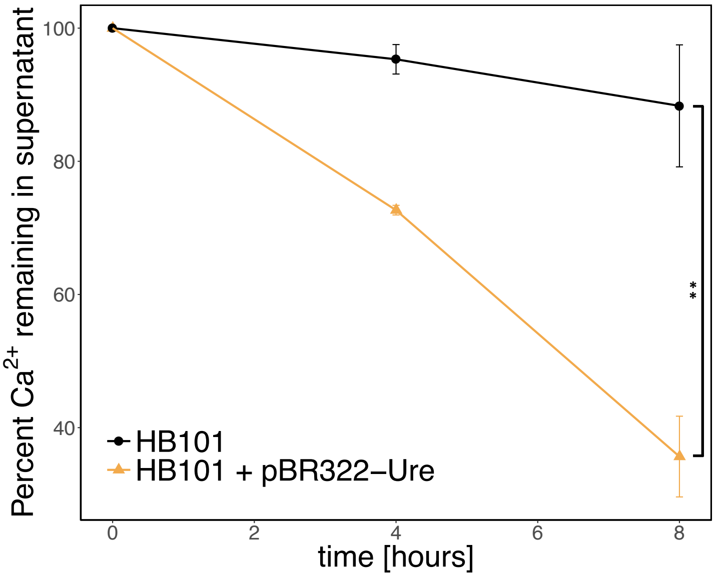

Figure S9. Soluble calcium levels in the culture supernatant during incubation of the capsules in urea and $CaCl_2$-containing media, normalized by initial calcium levels. Values and error bars reflect the mean ± s.d. of three biological replicates (n = 3). Calcium levels decreased significantly faster when the capsules contained the HB101/pBR322-Ure cells (comparison of values at 8-hour time point, two-sided Student *t*-test for two means, P-value = 0.0011). ** indicates P-value < 0.01.

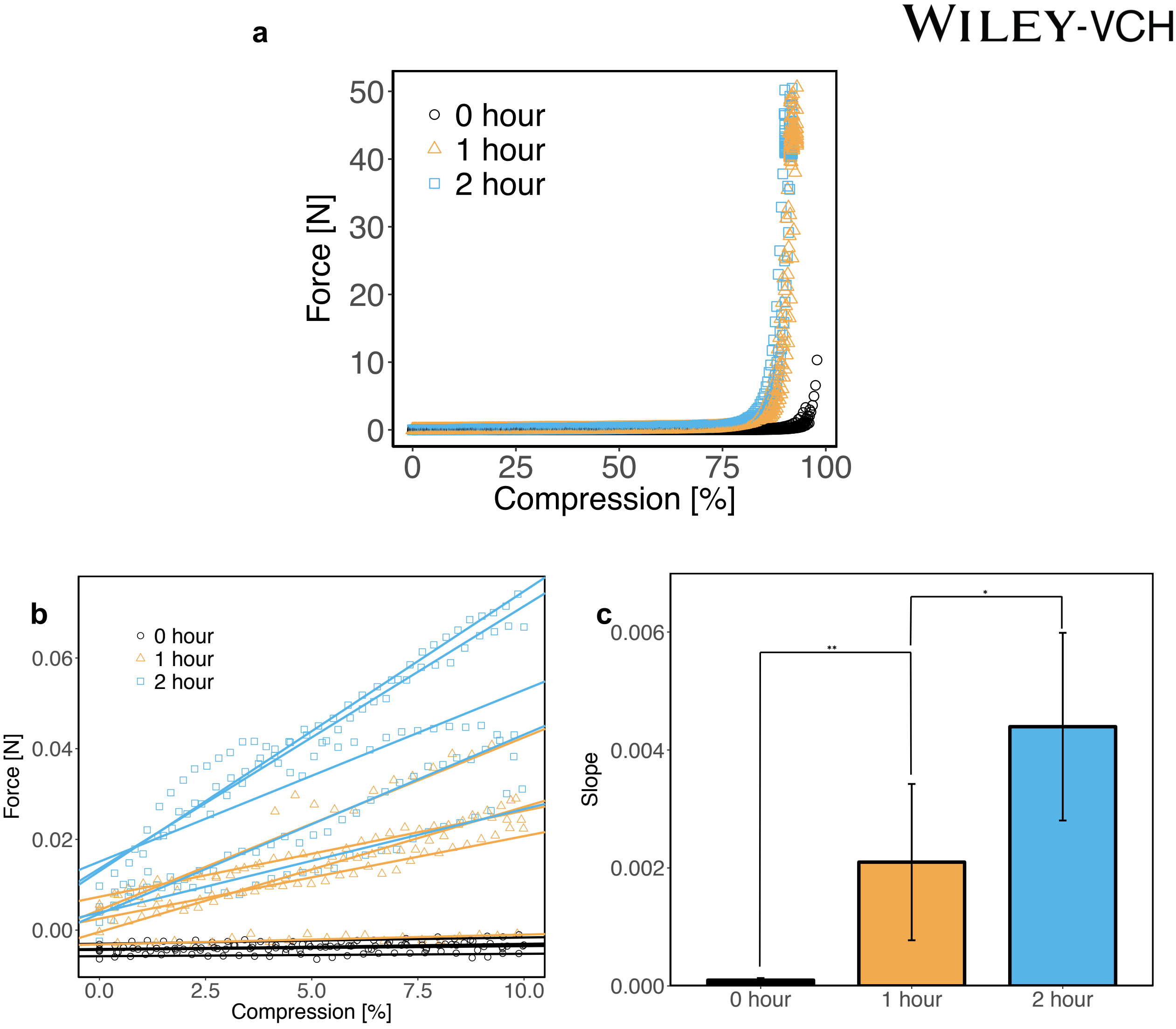

Figure S10. Data from compression testing of capsules containing HB101 cells with plasmid pBR322-Ure. In (a) and (b), the x-axis shows the normalized distance between the compression plates, where 0% represents the point of first contact between the capsule and top plate, and 100% represents touching of the compression plates. Capsules were incubated in urea- and $CaCl_2$-containing media for 0, 1, or 2 hours prior to testing. (a) The full range of data. (b) The same data as panel (a) after magnification, and with the addition of regression lines (computed in R) for each capsule in the displayed data range. (c) The average slopes of the regression lines. There is a significant increase in the average slope from the "0 hour" to "1 hour" capsules (two-sided Student *t*-test for two means, P-value = 0.0097, n = 5), and from the "1 hour" to "2 hour" capsules (two-sided Student *t*-test for two means, P-value = 0.0381, n = 5). ** indicates P-value < 0.01. * indicates P-value < 0.05.

Table S1. Strains used in this study.

**Strain**	**Description**	**Source**
BL21	Commercially available protein expression *E. coli* strain	Sigma-Aldrich CMC0016
PQN4	MC4100, ΔcsgBACEFG, λ(DE3), CamR	Dorval Courchesne et al.[17]
Gluconacetobacter hansenii	Acetic acid bacterium which produces high levels of BC	ATCC 53582
HB101	Commercially available protein expression *E. coli* strain	Promega L2015

Table S2. Plasmids used in this study.

**Plasmid**	**Description**	**Source**
pBbA8k-RFP	Arabinose-inducible expression of mRFP1	Addgene # 35273
pBbB8k-csg-WT	Arabinose-inducible expression of the full *csg* operon. *Csg* operon inserted in place of GFP in Addgene # 35363**.**	This study
pBbB8k-csg-NbGFP	Arabinose-inducible expression of the full *csg* operon in which *csgA* is fused to NbGFP, a VHH domain specific for GFP, by a 14aa GS linker.	This study. Available at Addgene # 166858.

pBbB8k-csg-NbStx2	Arabinose-inducible expression of the full *csg* operon in which *csgA* is fused to NbStx2, a VHH domain specific for shiga toxin 2, by a 14aa GS linker.	This study
pBbB8k-csg-amylase	Arabinose-inducible expression of the full *csg* operon in which is *csgA* fused to α-amylase from *Bacillus licheniformis* by a 14aa GS linker.	This study. Available at Addgene # 166859.
pLO7	Constitutive expression of *luxCDABEGH* operon from *V. harveyii*.	This study
pBR322-Ure	Urease gene cluster from *S. pasteurii*.	Liang et al.[48]
